# Selective depletion of regulatory T cells enhances the immunogenicity of a recombinant-based vaccine against *Sporothrix* spp

**DOI:** 10.3389/fcimb.2022.1084526

**Published:** 2023-02-10

**Authors:** Alexander Batista-Duharte, Damiana Téllez-Martínez, Deivys Leandro Portuondo, Iracilda Zeppone Carlos

**Affiliations:** Department of Clinical Analysis, School of Pharmaceutical Sciences, São Paulo State University (UNESP), Araraquara, SP, Brazil

**Keywords:** regulatory T cells, *Sporothrix schenckii*, vaccine, enolase, sporotrichosis, DEREG mice

## Abstract

**Introduction:**

Regulatory T cells (Tregs) have been shown to limit the protective immune response against pathogenic species of the fungus *Sporothrix* spp, the causal agent of sporotrichosis. However, the specific function of Tregs during vaccination against these fungi is known.

**Methods:**

We evaluated the effect of Tregs depletion on the immunogenicity of an experimental recombinant anti-*Sporothrix* vaccine, using the DEREG mice. In this model, only Foxp3(+) Tregs express eGFP and diphtheria toxin (DT) receptors, and transient Tregs depletion is achieved by DT administration.

**Results:**

Tregs depletion enhanced the frequency of specific IFNγ+ T cells (Th1 lymphocytes) and cytokine production after either the first or second vaccine dose. However, depletion of Tregs during the second dose caused greater stimulation of specific Th1 lymphocytes than depletion during the first dose. Similarly, the highest production of IgG, IgG1, and IgG2a anti rSsEno antibody was detected after Tregs depletion during boost immunization compared to the other immunized groups. Importantly, vaccine immunogenicity improvement after Tregs depletion also had an impact on the more efficient reduction of fungal load in the skin and liver after the challenge with *S. brasiliensis* in an experimental infection model. Interestingly, the reduction in fungal load was greatest in the Tregs depleted group during boosting.

**Discussion:**

Our results illustrate that Tregs restrict vaccine-induced immune response and their transient depletion could enhance anti-*Sporothrix* vaccine immunogenicity. Further studies are required to elucidate whether Tregs depletion may be a way to improve the efficacy of vaccination against *Sporothrix* spp.

## Introduction

Regulatory T cells (Tregs) are a subset of specialized CD4+T lymphocytes that express the transcription factor Foxp3 and play an important role in controlling immune responses in cancer, infectious and autoimmune diseases (Sakaguchi et al., 2010). In addition to Foxp3, classic Tregs have been characterized by high expression of the activation marker CD25 (Sakaguchi et al., 1995) and in humans, by the low levels of the alpha chain of Interleukin-7 (IL-7) receptor, CD127 (Hartigan-O’Connor et al., 2007). Tregs can interfere with the adequate induction of the immune response if their activity is increased during the induction of the immune response (Qin et al., 2015). Tregs-mediated immunosuppression may be due to contact-dependent (cell-cell direct interactions) and contact-independent mechanisms (mediated by immunosuppressive soluble factors (Schmidt et al., 2012).

Since the discovery of Tregs, there has been a growing interest in understanding how they can influence the response to vaccines to design more effective vaccine formulations. Experimental evidence suggests that Tregs are involved, at least in part, in the low efficacy of vaccines against various diseases where it has still been difficult to obtain protective immunization. When Tregs are depleted with monoclonal anti-CD25 antibodies, it is possible to observe greater immunogenicity against vaccine antigens (Jaron et al., 2008; Bright et al., 2013; Qin et al., 2015). Vaccine immunogenicity improvement by Tregs depletion has also been demonstrated in other models targeting Foxp3 (Batista-Duharte et al., 2018a; Mousavi-Niri et al., 2019).

Currently, there is great interest in developing vaccines against fungi, due to the elevated mortality of systemic mycosis in immunocompromised patients. Despite efforts to obtain antifungal vaccines, none are yet in clinical use, although many are in experimental phases (Pattison et al., 2021). Currently, there are different vaccine candidates against sporotrichosis, an emergent worldwide subcutaneous mycosis that is considered endemic in different tropical and subtropical countries tropical and subtropical countries although it has been increasingly observed in other regions (Téllez-Martínez et al., 2019a). The disease is caused by at least four species of the genus *Sporothrix*, including *Sporothrix schenckii sensu stricto* and *Sporothrix brasiliensis* the two most important pathogenic species*, Sporothrix globosa*, described mainly in Asia and *Sporothrix luriei* that cause sporadic cases (de Beer et al., 2016 *). S. brasiliensis*, is the most virulent species, involved in an expanding zoonosis transmitted by cats in Brazil (Gremião et al., 2017).

Recently, we evaluated the role of Tregs in *S. schenckii* and *S. brasiliensis* infection and observed an increased Tregs frequency associated with elevated fungal load in the skin and systemically in chronic infection (Batista-Duharte et al., 2018b). Importantly, the depletion of Tregs promoted faster fungal clearance, associated with enhanced Th1/Th17 response (Batista-Duharte et al., 2020). However, the role of Tregs in the efficacy of antifungal vaccines has not been sufficiently evaluated.

We, therefore, aimed to determine the role of Tregs in the immunogenicity and protective immunity of a recombinant enolase vaccine against *Sporothrix* spp. Here, we used the DEREG mice, a transgenic model in which Foxp3+ Tregs can be specifically depleted to study the specific function of these cells during vaccination. DEREG mice express a diphtheria toxin (DT) receptor-eGFP fusion protein under the control of the Foxp3 gene locus and Foxp3+ Tregs are depleted after DT injection (Lahl and Sparwasser, 2011). 

## Materials and methods

### Mouse strains and ethics statement

Male 6- to 8-week-old wild-type (WT) C57BL6 and C57BL/6DTR/eGFP (DEREG) mice were used in this study. WT C57BL6 mice were purchased from Centro Multidisciplinar para Investigacão Biologica na Area da Ciencia de Animais de Laboratorio” (CEMIB), University of Campinas (UNICAMP). DEREG mice were purchased with a collaboration transfer from Prof. Vera Lucia Garcia Calich, from the Institute of Biomedical Sciences of the University of Sao Paulo. All animal procedures were approved by the Ethics Committee for Animal Use in Research of Araraquara´s School of Pharmaceutical Sciences, UNESP, (Protocol CEUA/FCF/CAR no. 08/2016) and were performed according to the guidelines of the Brazilian College of Animal Experimentation (COBEA).

### Recombinant *S. schenckii* enolase (rSsEno) vaccine preparation

The recombinant *S. schenckii* enolase used as an antigen in this study was obtained and characterized as previously described (Portuondo et al., 2019). The vaccine formulation was prepared by mixing 100 µg of rSsEno with 5% Montanide Gel01, kindly provided by Seppic (Paris, France). 

### Immunization schedule and depletion of Tregs

Both male WT littermate and DEREG mice received two subcutaneous (s.c) injections (0.1 ml) on the back of the neck on days 0 and 14 with rSsEno + Gel 01, or PBS alone as the non-immunized control. Tregs depletion in DEREG mice was performed as previously described (Lahl and Sparwasser, 2011). Briefly, DEREG mice were treated with diphtheria toxin (DT) (Merck, catalog number 322326). 1 µg of DT in 100 µL of PBS was administered intraperitoneally (i.p.) into each mouse for two consecutive days, before 24 hours and at the moment of the immunization. One group received CT treatment during the first dose (priming) and another group during the second dose (booster). Vaccinated WT littermate mice received CT treatment during the booster. Blood samples were collected from the tail vein immediately before the first DT administration, at 48 h, and on the 6th day after the second DT injection in each group of vaccinated mice, and eGFP+ Tregs % was measured by flow cytometry with a BD Accuri C6 flow cytometer (BD Biosciences).

### Preparation of splenocytes and *ex-vivo* stimulation

Mice (n=7 per group) were euthanized 21 days after primary immunization to evaluate the specific response of Th1/Th17 lymphocytes and antibodies. Each spleen was removed and homogenized, with 6 mL of a 0.17 M ammonium chloride solution and incubated on ice for 5 min for red cell lysis. The cell suspensions were centrifugated and, washed once with 3mL of RPMI-1640 (Sigma–Aldrich, Germany), supplemented with 2 mm l-glutamine, 100 U/mL penicillin, 100 µg/mL of streptomycin, and 10% fetal calf serum (RPMI complete) and then the splenocytes were resuspended in 1mL of the same medium. Cell concentration and cell viability were determined by microscopy using the Trypan blue exclusion test and viable splenocytes were adjusted to 2.5×10^6^ cells/mL in RPMI complete and conserved in ice for the following studies. The splenocytes were stimulated for 24 hours with 10µg of rSsEno/mL. Concanavalin A (0.25 µg/mL) or RPMI complete were used as either positive or negative controls, respectively.

### Flow cytometry immunophenotyping

The following mAbs were used: anti -CD16/CD32 purified (Mouse BD Fc Block™, unlabeled, clone 2.4G2, BD), anti -CD3 FITC (Fluorescein isothiocyanate) (clone 17A2), anti-CD4 APC (allophycocyanin) (clone RM4 -5), anti-CD25 PE (phycoerythrin) (clone PC61 -5), anti-Foxp3-PE-Cy7 (phycoerythrin-cyanine7) (clone FJK-16s), anti-IL-17-PE (clon eBio17B7) and anti-IFN-γ PerCP-Cy5.5, (all antibodies and their respective isotype controls were purchased from eBiosciences). Briefly, splenocytes were stained for the extracellular markers, then fixed and permeabilized using eBiosciences’ intracellular fixation (Thermo Scientific, Waltham, MA, USA) and permeabilization buffer set, and then the intracellular IFN-γ and IL-17A were stained with the fluorescent respective markers. Intracellular cytokines were detected after *in vitro* treatment with rSsEno and Brefeldin A, for intracellular retention the induced cytokines. Tregs were gated either as CD4+CD25+Foxp3+ in WT or CD4+CD25+Foxp3GP+ in DEREG mice. Th1 and Th17 populations were gated either as CD4+ IFNγ+ cells or CD4+ IL17A+ cells, respectively. The events were acquired using a BD Accuri C6 flow cytometer (BD Biosciences) and analyzed with the BD Biosciences software package. At least 50,000 events were effectively included in each analysis.

### Cytokines

The concentration of IL-2, IFN-γ, IL-6, IL-17 and IL-10 was measured in the supernatant of splenocytes previously stimulated during 24 hours with rSsEno (without Brefeldin A), using a BD™ cytometric bead array (BD Biosciences Th1/Th2/TH17 Cytokine Kit, Cat. No. 560485), according to the manufacturer’s instructions. The events were acquired with a BD Accuri C6 flow cytometer (BD Biosciences) and analyzed with the flow cytometer’s proprietary software.

### Serum anti- rSsEno IgG, IgG1 and IgG2a

The sera obtained two weeks after the second immunization were heat inactivated at 56°C for 30 minutes, aliquoted, and stored at -20°C. Serum levels of anti-rSsEno IgG, IgG1 and IgG2a were measured by ELISA as previously described (Portuondo et al., 2016).

### Microorganism and experimental infection

The *S. brasiliensis* ss250 strain (CBS 133009, GenBank: KC693883.1) kindly provided by the Oswaldo Cruz Foundation, Rio de Janeiro, Brazil, was isolated from feline sporotrichosis, Fungal preparation and experimental infection in DEREG mice at 21 days after the primary vaccination were performed as previously described (Batista-Duharte et al., 2020). Four group of animals (n=7 per group) (non-vaccinated, vaccinated with rSsEno + Gel 01 without Tregs depletion or depleted either during the first or the second doses), were used to investigate the influence of Tregs depletion on the fungal load at 35 days post-infection (d.p.i).

### Preparing tissue homogenates and fungal load assessment

Freshly isolated specimens of skin (site of primary infection) and liver (to evaluate systemic fungal dissemination), from each animal, were homogenized separately under sterile conditions and passed through a 100 µm cell strainer into a Petri dish containing 2mL of PBS with the aid of a syringe plunger. The fungal load was assessed by counting the CFU grown on Mycosel agar plates after the spread-plating of a previously determined dilution of the respective organ macerate.

### Statistical analysis

All statistics were performed using Graph Pad Prism version 8.0.2. Either two way analysis of variance (ANOVA) or one-way ANOVA followed by Tukey’s post-test were used according to the experimental design. Data from the CFU assay were analyzed by the Mann-Whitney U- test. The results are expressed as the mean ± SD. Statistically significant results are marked with *, **, *** or **** denoting p < 0.05, p < 0.01, p < 0.001 or p < 0.0001, respectively.

## Results

### Kinetics of Tregs depletion in vaccinated DEREG mice

**Figure 1:**
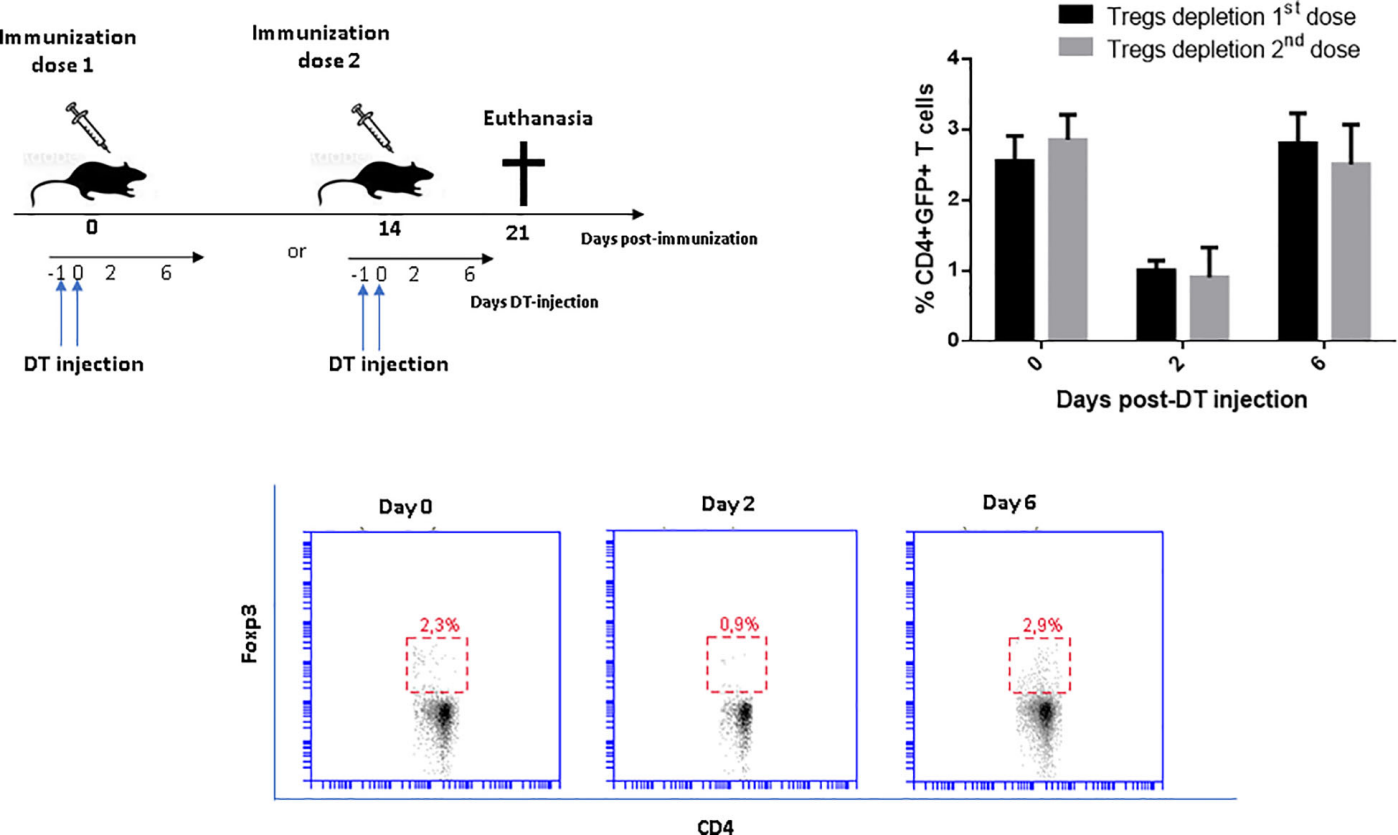
Kinetics of Tregs depletion in vaccinated DEREG mice with a recombinant-based vaccine against *Sporothrix* spp. The consecutive intraperitoneal application of DT over two days leads to a depletion of eGFP+Foxp3+ Tregs within 48 h and complete recovery around the sixth day of DT administration. The percentage of CD4+GFP+ Tregs in peripheral blood was measured by flow cytometry at the indicated time points. There were no differences in the magnitude of Tregs depletion at priming and boosting. Wild-type littermates mice also received similar DT treatment during the boosting without effects on Tregs frequency (results not shown). Two-way ANOVA analysis revealed significant differences (p<0.0001) on day 2 compared to days 0 and 6 after DT injection in both groups, with no difference between them at any time point.

eGFP+ Tregs were depleted by i.p. injection of DT in vaccinated DEREG mice during either the first or second dose. The frequency (%) of Tregs in the blood rapidly dropped from around 2,8% immediately before immunization, to less than 1% 48 h after DT injection. The number of Tregs was recovered by the 6^th^ day post-immunization (**Figure 1**). Depletion of Tregs in DEREG mice did not cause toxicity signs.

### Tregs depletion leads to *ex-vivo* expansion of Th1 lymphocytes in vaccinated mice

**Figure 2:**
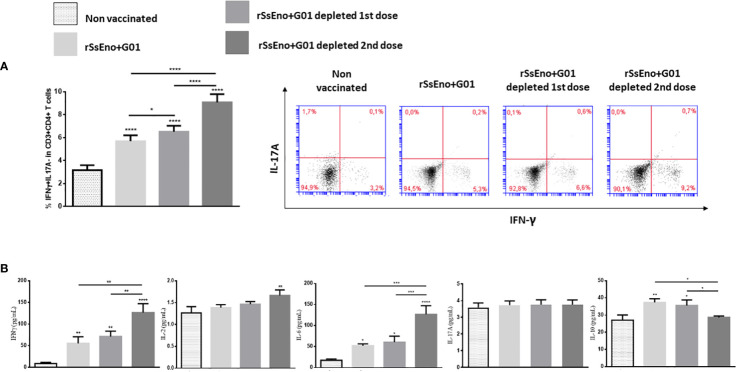
Tregs depletion during boost immunization leads to increased stimulation of IFNγ+Th1 lymphocytes **(A)**, and increased production of IFNγ, IL-2, and IL-6 associated with reduced IL-10 compared to the group without Tregs depletion **(B)**. Vaccination did not stimulate IL-17 production. To analyze the effect of Tregs depletion on the Th1/Th17 response, both male WT littermate and DEREG mice received two subcutaneous injections (0.1 ml) on days 0 and 14 with rSsEno + Gel 01, or PBS alone (non-vaccinated control). Immunized mice were treated with 1 μg of diphtheria toxin (DT) intraperitoneally (i.p.) for two consecutive days, before 24 hours, and during either the first (priming) or the second dose (booster). Immunized and control mice were euthanized 21 days after the primary immunization and their splenocytes were stimulated for 24 hours with 10μg of rSsEno/mL. The supernatant-accumulated cytokines (IL-2, IL-6, IL17A, IFN-γ, and IL-10) were measured by Cytometric Bead Array Th1/Th2/Th17 Cytokine Kit. The results are presented as the mean ± SD of 7 mice per group from two independent experiments, and statistical significance was determined by one-way ANOVA using Tukey’s multiple comparisons tests. *(p < 0.05); **(p < 0.01); ***(p < 0.001); ****(p < 0.0001).

Th1 lymphocytes play a prominent role in enabling antifungal mechanisms to control infections caused by *S. schenckii* and *S. brasiliensis *in infected individuals. In this study, we addressed the question of how Tregs depletion affects the frequency of Th1 lymphocytes following *ex-vivo* stimulation with rSsEno. An increased expansion of IFN-γ+ CD4+ T cells was seen in mice vaccinated with rSsEno + Gel 01 and Tregs-depleted after splenocytes stimulation with rSsEno. Those animals whose Tregs were depleted during the second-dose administration timeframe exhibited the strongest stimulation of Th1 lymphocytes (p<0.0001) with respect to the other groups (**Figure 2A**). These results were confirmed by measuring the concentration of different cytokines in the supernatant of splenocytes previously stimulated with rSsEno. Elevated production of IFN-γ (p<0.0001), IL-2 (p<0.01) and IL-6 (p<0.0001) was also detected in the group with Tregs depleted during boost vaccination. Interestingly, reduced production of IL-10 was also observed in that group (p< 0.05 compared to the vaccinated/non-Tregs depleted group) (**Figure 2B**). Immunization did not change IL-17 concentrations in either group.

### Anti- rSsEno antibody production is enhanced in vaccinated Tregs-depleted mice

**Figure 3:**
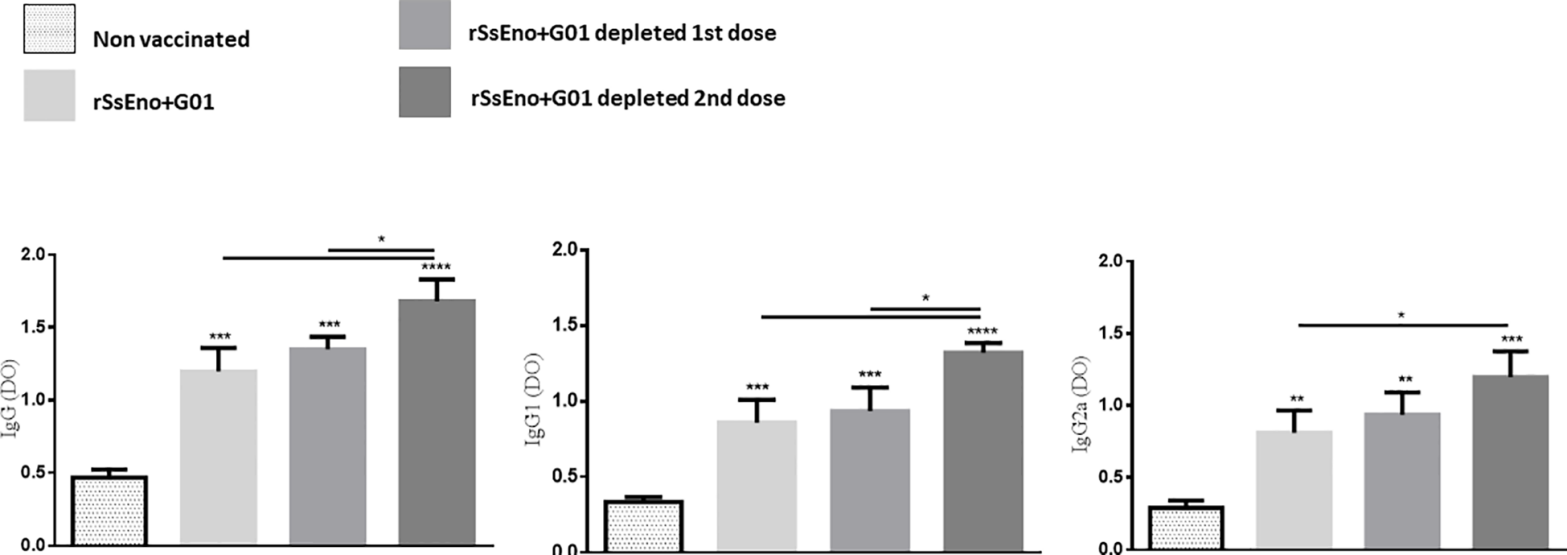
Tregs depletion during anti rSsEno booster vaccination promotes enhanced production of serum IgG, IgG1 and IgG2a specific antibodies. *(p < 0.05); **(p < 0.01); ***(p < 0.001); ****(p < 0.0001).

To analyze the effect of Tregs depletion on the production of specific antibodies, the titer of specific IgG, IgG1, and IgG2a was quantified in the serum extracted from the animals’ blood samples. As depicted in **Figure 3**, the groups immunized and Tregs-depleted, produced higher titers of specific antibodies than non-Tregs-depleted mice, with statistically significant differences in the depleted groups during the administration of the second dose (p < 0.05).

### Transient Tregs depletion during immunization favors the fungal clearance in S. brasiliensis-infected mice

**Figure 4:**
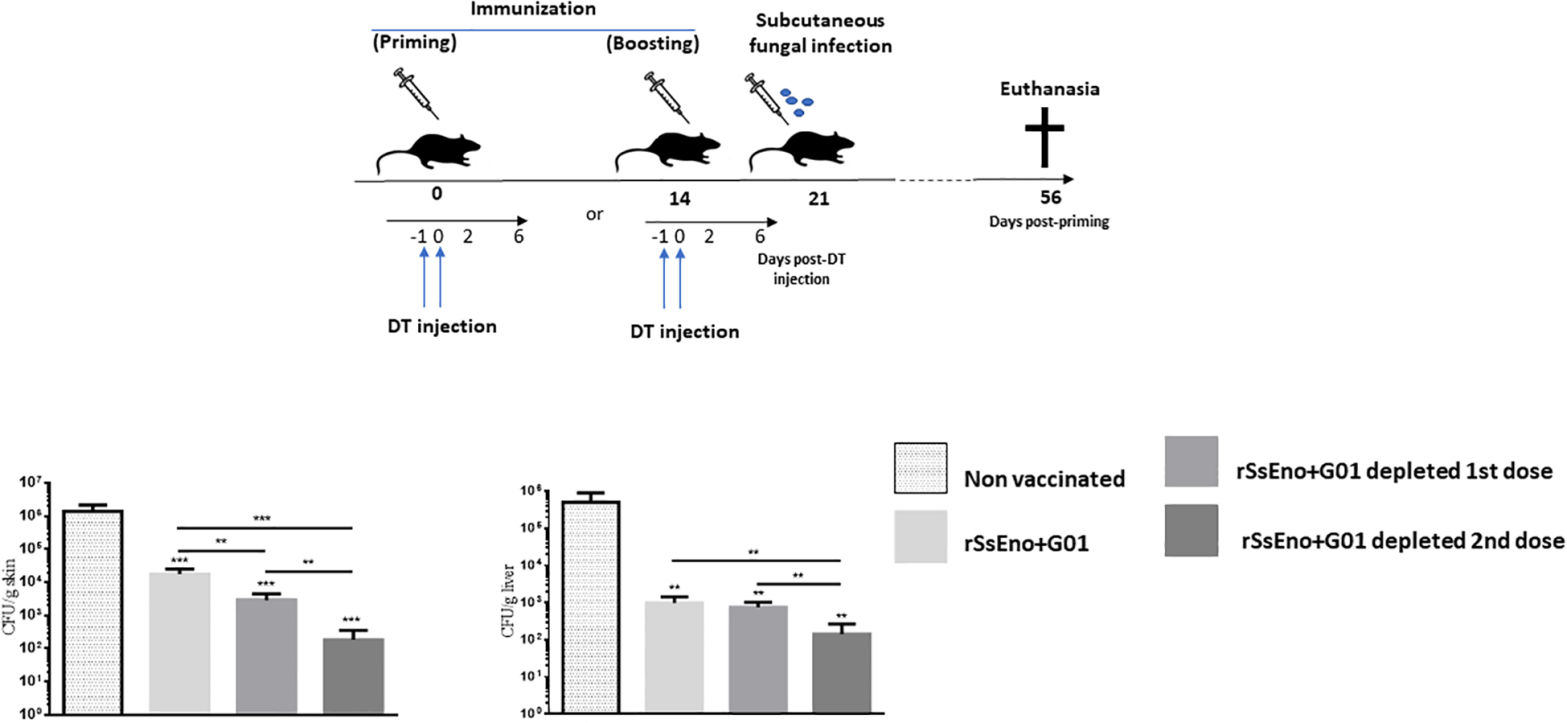
Tregs-depletion during boost immunization confers enhanced protection against a challenge with *S. brasiliensis* conidia compared with non-depleted Tregs vaccinated mice and those depleted during the prime vaccination. To analyze the effect of Tregs depletion on protection against the fungal infection in vaccinated mice, wild-type littermates and DEREG mice were immunized on days 0 and 14 with a recombinant-based vaccine against *Sporothrix* spp. On days -1, and 0, all mice were treated with DT. At day 21 post priming all mice were challenged with 1 x10^7^ conidia by subcutaneous injection. The resulting fungal load was monitored at 56 days post-priming at the site of inoculation and in the liver (local and systemic dissemination respectively). This experiment was repeated twice with similar results. **(p < 0.01); ***(p < 0.001).

Given the above results, we sought to examine the effects of Tregs depletion during either the first or second vaccination, on fungal clearance in the skin and systemically in infected mice at 35 d.p.i. As shown in **Figure 4**, infected mice previously vaccinated and Tregs depleted had a lower fungal burden in the skin and subcutaneous tissue as well as in the liver than non-vaccinated and vaccinated mice that did not receive treatment with DT for Tregs depletion. Notably, the group with Tregs depletion during the second dose of the vaccine exhibited a reduced skin ulcer and lower fungal load in the skin (p < 0.001) and in the liver (p < 0.01), compared with the other groups.

Clinical observation of the animals during the infection period revealed that immunized and Treg-depleted mice showed fewer suppurative ulcerative primary lesions on the skin than non-immunized and even non-depleted vaccinated mice. In addition, yellowish-white nodules in the liver were scarcely present in vaccinated mice, compared to non-vaccinated mice (**Supplementary Data**).

## Discussion

The beneficial effect of Tregs depletion as a way to control different types of fungal infections has been demonstrated (Felonato et al., 2012; Whibley et al., 2014; Galdino et al., 2018; Batista-Duharte et al., 2020). In contrast, the use of this strategy to improve preventive vaccines has been less studied. Anti-CD25 treatment elicits Tregs depletion and enhances vaccine-induced T cell responses (Rech and Vonderheide, 2009; Klages et al., 2010; Tang et al., 2017; Wen et al., 2017). However, this approach is not specific since there are populations of T cells that are not Tregs, which express CD25 during activation (Couper et al., 2009; Onda et al., 2019). The discovery of Foxp3 as a more specific marker of Tregs (Fontenot et al., 2003; Hori et al., 2003), opened the doors to the search for new modulation tools based on the silencing of this gene for the improvement of specific immune responses (Mousavi-Niri et al., 2019). What is challenging about this strategy is that Tregs depletion, as with other approaches, must be transient to avoid severe autoimmune and inflammatory reactions (Batista-Duharte et al., 2018a). 

In preclinical studies, using the DEREG mouse it is possible to temporarily disable the Foxp3 +Tregs function by their depletion at selected time points by means of DT treatment (Lahl and Sparwasser, 2011). After DT injection, on two or three consecutive days, around 95% of CD4+ GFP-Foxp3+ Tregs can be specifically depleted, but after stopping DT injection, their levels recover quickly (Mayer et al., 2014). The rapid rebound of Tregs in DEREG mice prevents inflammatory and autoimmunity reactions (Berod et al., 2014). Thus, the DEREG mouse is an ideal model to study specifically the influence of Tregs vaccine-induced immune response. In this study, we used a recommended schedule for Tregs depletion with DT injections for two consecutive days (Lahl and Sparwasser, 2011), during the administration of either the first or second dose of the vaccine. As expected, using this protocol the Tregs percentage dropped significantly and was recovered by the 6th day after injection. With this transitory inhibition, we generated a time window of a few days where the numbers of Tregs were significantly reduced and thus we could evaluate their effects on the vaccine-induced immune response. Similar strategies with DEREG mice to evaluate the effect of other anti-infectious vaccines have previously been described (Espinoza et al., 2014; Berod et al., 2014).

Herein, we emphasized the response of Th1/Th17 cells due to their proven role in controlling *S. schenckii* and *S. brasiliensis* infection. The Th1-mediated response is driven by the release of IFNγ, which favors the stimulation of macrophages, opsonophagocytosis, and the release of fungicidal mechanisms (Maia et al., 2006; Maia et al., 2016). Besides, the Th17 response stimulates the infiltration of neutrophils and evidence suggests that it is also involved in fungal clearance (Ferreira et al., 2015; Batista-Duharte et al., 2018b; Batista-Duharte et al., 2020). Upon depletion of Tregs during priming, we found an enhanced number of rSsEno-specific T cells producing IFNγ after *in vitro* stimulation with rSsEno in vaccinated mice. The highest elevation of CD4+IFNγ+ T cells was observed in Tregs-depleted mice, mainly in the second dose of the vaccine. This increase in IFNγ+ T cells was associated with elevated production of IL-2, IFNγ, and IL-6 in the supernatant of splenocytes stimulated *in vitro* with the vaccine antigen. In contrast, lower production of IL-10 was detected in the group with Tregs depletion during boost vaccination. Previous studies have reported that Tregs elicit the downregulation of the costimulatory molecules CD80, CD83, CD86, and CD40 in dendritic cells (DC) (Misra et al., 2004). This effect is associated with the up-regulation of IL-10 (Veldhoen et al., 2006) and the coinhibitory molecule B7-H3 (also known as CD276) (Mahnke et al., 2007). Other studies reported that DT-mediated depletion of Foxp3+ cells in DEREG mice induced increased division of DC and precursor DC in lymphoid organs and up-regulation of CD80, CD86 and CD40 in the context of autoimmunity (Schildknecht et al., 2010). More recently, it was demonstrated that antigen-specific Tregs remove pMHCII complexes from the DC surface thus decreasing the activity of DCs as antigen-presenting cells (Akkaya et al., 2019). Thus, Tregs might limit DC maturation by preventing signal strength, consequently reducing T cells’ priming. In this study, the magnitude of Tregs depletion was similar in both doses but the strongest effect of Tregs depletion was observed after the second dose with a significant effect on the IFNγ production by activated T lymphocytes with rSsEno. In this case, we consider that the higher production of IFNγ, induced a reduction of IL-10 in other populations included in splenocytes (e.g. B and T lymphocytes, macrophages, dendritic cells) (Hu et al., 2006; Saraiva and O’Garra, 2010). On the other hand, no significant stimulation of the Th17-mediated immune response in vaccinated mice was observed in this study, and Tregs depletion did not change this pattern. In other previous studies with this same vaccine formulation, we had already observed that it did not stimulate a significant Th17 response (Portuondo et al., 2019; Téllez-Martínez et al., 2019b).

A similar result to the Th1 response was observed in the production of anti rSsEno antibodies. The highest production of specific IgG, IgG1, and IgG2a antibodies was detected after the depletion of Tregs during boost immunization compared to the other immunized groups (Batista-Duharte et al., 2020). In a recent study, Jacobsen et al. (2021), demonstrated that contraction of the germinal center (GC) in lymph nodes induced by immunization is immediately preceded by a wave of Foxp3+ T cells in GC, attributed at least partly to up-regulation of Foxp3 by T follicular helper (TFH) cells. Our study did not delve into the events that occur in the post-immunization germinal center, but we believe that this phenomenon described by Jacobsen et al. could be involved, in the stimulation of antibody production after immunization and Tregs depletion. Further studies are required to elucidate this effect.

To evaluate the impact of increased vaccine immunogenicity due to Tregs depletion in both the first and second doses, we used an experimental model of subcutaneous infection that has already been used by our group in studies of Tregs depletion in DEREG mice (Batista-Duharte et al., 2020). Immunized DT-treated DEREG mice and wild-type littermates were challenged at day 14 post-boosting by subcutaneous injection of *S. brasiliensis* conidia. A non-immunized wild-type littermates group was used as a control. Tregs depletion during priming and most notably during boosting favored the faster fungal clearance in our model. In a previous study, it was shown that Tregs depletion during boost immunization leads to reduced parasitic burden after challenge with *P. berghei* sporozoites (Espinoza et al., 2014). These authors also demonstrated that Tregs depletion during prime immunization confers only partial protection against a challenge with *P. berghei* sporozoites. These results support the fact that not only priming but also lymphocyte expansion after boosting is strongly influenced by Tregs. 

In summary, our results provide evidence that transient depletion of Tregs could enhance a vaccine-induced response during prime and boost immunization. Importantly, the enhanced expansion of T effector cells and antibody response after Tregs depletion was accompanied by increased protection against the fungal infection. All this suggests that the modulation of the immunosuppressive activity of Tregs could be a way of improving vaccines against *Sporothrix* spp. Further studies are required to unravel the exact function of Tregs throughout the vaccination process and the effect of their transient depletion on vaccine efficacy, immunological memory, and long-term toxicity.

## Data availability statement

The raw data supporting the conclusions of this article will be made available by the authors, without undue reservation.

## Ethics statement

The animal study was reviewed and approved by Ethics Committee for Animal Use in Research of Araraquara´s School of Pharmaceutical Sciences, UNESP, (Protocol CEUA/FCF/CAR no. 08/2016).

## Author contributions

AB-D and DT-M contributed to conception and design of the study; AB-D, DT-M, and DP investigation, resources, data curation; AB-D and DT-M performed the statistical analysis; AB-D wrote the first draft of the manuscript; AB-D, DT-M, and DP wrote sections of the manuscript; IZ review & editing, supervision, formal analysis. All authors contributed to the article and approved the submitted version.

## Supplementary material

Supplementary Figure 1 Three aspects of primary lesions in the dorsal sacral region of mice infected with *S. brasiliensis.* **(A)** Suppurative ulcer; **(B)** Nodular lesion with a little central ulceration; **(C)** Nodular lesion.

Supplementary Table 1 Main clinical findings in the *S. brasiliensis-* infected mice at 35 days post infection. *In vaccinated mice the nodules in the liver were scarcely present, in a small number of animals. Morphological characteristic of the yellowish-white nodules in the liver of *S. brasiliensis-* infected mice, can be seen in (Batista-Duharte et al., 2018b).

## References

[B1] Akkaya B. Oya Y. Akkaya M. Al Souz J. Holstein A. H. Kamenyeva O.. (2019). Regulatory T cells mediate specific suppression by depleting peptide-MHC class II from dendritic cells. Nat. Immunol. 20 (2), 218–231. doi: 10.1038/s41590-018-0280-2 30643268 PMC6402611

[B42] Solution to a problematic relationship. Stud. Mycol. 83, 165e91. doi: 10.1016/j.simyco.2016.07.001 PMC5007658 27616802

[B2] Batista-Duharte A. Téllez-Martínez D. de Andrade C. Polesi M. C. Portuondo D. L. Carlos I. Z.. (2020). Transient Foxp3(+) regulatory T-cell depletion enhances protective Th1/Th17 immune response in murine sporotrichosis caused by *Sporothrix schenckii* . Immunobiology 225 (5), 151993. doi: 10.1016/j.imbio.2020.151993 32962813

[B4] Batista-Duharte A. Téllez-Martínez D. de Andrade C. Portuondo D. L. Jellmayer J. A. Polesi M. C.. (2018b). *Sporothrix brasiliensis* induces a more severe disease associated with sustained Th17 and regulatory T cells responses than *Sporothrix schenckii sensu stricto* in mice. Fungal Biol. 122 (12), 1163–1170. doi: 10.1016/j.funbio.2018.08.004 30449354

[B3] Batista-Duharte A. Téllez-Martínez D. Fuentes D. L. P. Carlos I. Z. (2018a). Molecular adjuvants that modulate regulatory T cell function in vaccination: A critical appraisal. Pharmacol. Res. 129, 237–250. doi: 10.1016/j.phrs.2017.11.026 29175113

[B5] Berod L. Stüve P. Varela F. Behrends J. Swallow M. Kruse F.. (2014). Rapid rebound of the treg compartment in DEREG mice limits the impact of treg depletion on mycobacterial burden, but prevents autoimmunity. PloS One 9 (7), e102804. doi: 10.1371/journal.pone.0102804 25050936 PMC4106855

[B6] Bright J. D. Schultz H. N. Byrne J. A. Bright R. K. (2013). Injection site and regulatory T cells influence durable vaccine-induced tumor immunity to an over-expressed self-tumor-associated antigen. Oncoimmunology 2 (7), e25049. doi: 10.4161/onci.25049 24073379 PMC3782160

[B7] Couper K. N. Lanthier P. A. Perona-Wright G. Kummer L. W. Chen W. Smiley S. T.. (2009). Anti-CD25 antibody-mediated depletion of effector T cell populations enhances susceptibility of mice to acute but not chronic toxoplasma gondii infection. J. Immunol. 182, 3985–3994. doi: 10.4049/jimmunol.0803053 19299696 PMC3942880

[B8] de Beer Z. W. Duong T. A. Wingfield M. J. (2016). The divorce of *Sporothrix* and *Ophiostoma*. Solution to a problematic relationship. Stud. Mycol. 83, 165e91. doi: 10.1016/j.simyco.2016.07.001 27616802 PMC5007658

[B9] Espinoza M. R. Steeg C. Tartz S. Heussler V. Sparwasser T. Link A.. (2014). Depletion of regulatory T cells augments a vaccine induced T effector cell response against the liver-stage of malaria but fails to increase memory. PloS One 9 (8), e104627. doi: 10.1371/journal.pone.0104627 25115805 PMC4130546

[B10] Felonato M. Pina A. de Araujo E. F. Loures F. V. Bazan S. B. Feriotti C.. (2012). Anti-CD25 treatment depletes treg cells and decreases disease severity in susceptible and resistant mice infected with *Paracoccidioides brasiliensis* . PloS One 7 (11), e51071. doi: 10.1371/journal.pone.0051071 23226464 PMC3511355

[B11] Ferreira L. S. Gonçalves A. C. Portuondo D. L. Maia D. C. Placeres M. C. Batista-Duharte A.. (2015). Optimal clearance of *Sporothrix schenckii* requires an intact Th17 response in a mouse model of systemic infection. Immunobiology 220 (8), 985–992. doi: 10.1016/j.imbio.2015.02.009 25776919

[B12] Fontenot J. D. Gavin M. A. Rudensky A. Y. (2003). Foxp3 programs the development and function of CD4+CD25+ regulatory T-cells. Nat. Immunol. 4 (4), 330–336. doi: 10.1038/ni904 12612578

[B13] Galdino N. A. L. Loures F. V. de Araújo E. F. da Costa T. A. Preite N. W. Calich V. L. G. (2018). Depletion of regulatory T cells in ongoing paracoccidioidomycosis rescues protective Th1/Th17 immunity and prevents fatal disease outcome. Sci. Rep. 8 (1), 16544. doi: 10.1038/s41598-018-35037-8 30410119 PMC6224548

[B14] Gremião I. D. F. Miranda L. H. M. Reis E. G. Rodrigues A. M. Pereira S. A. (2017). Zoonotic epidemic of sporotrichosis: cat to human transmission. PloS Pathog. 13 (1), e1006077. doi: 10.1371/journal.ppat.1006077 28103311 PMC5245785

[B15] Hartigan-O’Connor D. J. Poon C. Sinclair E. McCune J. M. (2007). Human CD4+ regulatory T cells express lower levels of the IL-7 receptor alpha chain (CD127), allowing consistent identification and sorting of live cells. J. Immunol. Methods 319 (1-2), 41–52. doi: 10.1016/j.jim.2006.10.008 17173927

[B16] Hori S. Nomura T. Sakaguchi S. (2003). Control of regulatory T-cell development by the transcription factor Foxp3. Science 299 (5609), 1057–1061. doi: 10.1126/science.1079490 12522256

[B17] Hu X. Paik P. K. Chen J. Yarilina A. Kockeritz L. Lu T.. (2006). IFN-gamma suppresses IL-10 production and synergizes with TLR2 by regulating GSK3 and CREB/AP-1 proteins. Immunity 24 (5), 563–574. doi: 10.1016/j.immuni.2006.02.014 16713974

[B18] Jacobsen J. T. Hu W. Castro T. B. Solem S. Galante A. Lin Z.. (2021). Expression of Foxp3 by T follicular helper cells in end-stage germinal centers. Science 373 (6552), eabe5146. doi: 10.1126/science.abe5146 34437125 PMC9007630

[B19] Jaron B. Maranghi E. Leclerc C. Majlessi L. (2008). Effect of attenuation of treg during BCG immunization on anti-mycobacterial Th1 responses and protection against *Mycobacterium tuberculosis* . PloS One 3 (7), e2833. doi: 10.1371/journal.pone.0002833 18665224 PMC2475666

[B20] Klages K. Mayer C. T. Lahl K. Loddenkemper C. Teng M. W. Ngiow S. F.. (2010). Selective depletion of Foxp3+ regulatory T cells improves effective therapeutic vaccination against established melanoma. Cancer Res. 70 (20), 7788–7799. doi: 10.1158/0008-5472.CAN-10-1736 20924102

[B21] Lahl K. Sparwasser T. (2011). *In vivo* depletion of FoxP3+ tregs using the DEREG mouse model. Methods Mol. Biol. 707, 157–172. doi: 10.1007/978-1-61737-979-6_10 21287334

[B24] Mahnke K. Ring S. Johnson T. S. Schallenberg S. Schönfeld K. Storn V.. (2007). Induction of immunosuppressive functions of dendritic cells *in vivo* by CD4+CD25+ regulatory T cells: role of B7-H3 expression and antigen presentation. Eur. J. Immunol. 37 (8), 2117–2126. doi: 10.1002/eji.200636841 17615586

[B25] Maia D. C. Gonçalves A. C. Ferreira L. S. Manente F. A. Portuondo D. L. Vellosa J. C.. (2016). Response of cytokines and hydrogen peroxide to *Sporothrix schenckii* exoantigen in systemic experimental infection. Mycopathologia 181 (3-4), 207–215. doi: 10.1007/s11046-015-9966-2 26603044

[B26] Maia D. C. Sassá M. F. Placeres M. C. Carlos I. Z. (2006). Influence of Th1/Th2 cytokines and nitric oxide in murine systemic infection induced by *Sporothrix schenckii* . Mycopathologia 161 (1), 11–19. doi: 10.1007/s11046-005-0142-y 16389479

[B27] Mayer C. T. Ghorbani P. Kühl A. A. Stüve P. Hegemann M. Berod L.. (2014). Few Foxp3^+^ regulatory T cells are sufficient to protect adult mice from lethal autoimmunity. Eur. J. Immunol. 44 (10), 2990–3002. doi: 10.1002/eji.201344315 25042334

[B28] Misra N. Bayry J. Lacroix-Desmazes S. Kazatchkine M. D. Kaveri S. V. (2004). Cutting edge: Human CD4+CD25+ T cells restrain the maturation and antigen-presenting function of dendritic cells. J. Immunol. 172, 4676–4680. doi: 10.4049/jimmunol.172.8.4676 15067041

[B29] Mousavi-Niri N. Naseroleslami M. Hadjati J. (2019). Anti-regulatory T cell vaccines in immunotherapy: focusing on FoxP3 as target. Hum. Vaccin. Immunother. 15 (3), 620–624. doi: 10.1080/21645515.2018.1545625 30633616 PMC6605713

[B30] Onda M. Kobayashi K. Pastan I. (2019). Depletion of regulatory T cells in tumors with an anti- CD25 immunotoxin induces CD8 T cell-mediated systemic antitumor immunity. Proc. Natl. Acad. Sci. U.S.A. 116 (10), 4575–4582. doi: 10.1073/pnas.1820388116 30760587 PMC6410866

[B31] Pattison H. T. Millar B. C. Moore J. E. (2021). Fungal vaccines. Br. J. BioMed. Sci. 78 (4), 167–176. doi: 10.1080/09674845.2021.1907953 33751908

[B33] Portuondo D. L. Batista-Duharte A. Ferreira L. S. Martínez D. T. Polesi M. C. Duarte R. A.. (2016). A cell wall protein-based vaccine candidate induce protective immune response against *Sporothrix schenckii* infection. Immunobiology 221 (2), 300–309. doi: 10.1016/j.imbio.2015.10.005 26547105

[B34] Portuondo D. L. Dores-Silva P. R. Ferreira L. S. de Oliveira C. S. Téllez-Martínez D. Marcos C. M.. (2019). Immunization with recombinant enolase of sporothrix spp. (rSsEno) confers effective protection against sporotrichosis in mice. Sci. Rep. 9 (1), 17179. doi: 10.1038/s41598-019-53135-z 31748544 PMC6868355

[B35] Qin L. Jiang G. Han J. Letvin N. L. (2015). Regulatory T cells modulate DNA vaccine immunogenicity at early time *via* functional CD4(+) T cells and antigen duration. Front. Immunol. 6. doi: 10.3389/fimmu.2015.00510 PMC4586510 26483796

[B36] Rech A. J. Vonderheide R. H. (2009). Clinical use of anti-CD25 antibody daclizumab to enhance immune responses to tumor antigen vaccination by targeting regulatory T cells. Ann. N. Y. Acad. Sci. 1174, 99–106. doi: 10.1111/j.1749-6632.2009.04939.x 19769742

[B37] Sakaguchi S. Miyara M. Costantino C. M. Hafler D. A. (2010). FOXP3+ regulatory T cells in the human immune system. Nat. Rev. Immunol. 10, 490–500. doi: 10.1038/nri2785 20559327

[B38] Sakaguchi S. Sakaguchi N. Asano M. Itoh M. Toda M. (1995). Immunologic self-tolerance maintained by activated T cells expressing IL-2 receptor alpha-chains (CD25). breakdown of a single mechanism of self-tolerance causes various autoimmune diseases. J. Immunol. 155 (3), 1151–1164. 7636184

[B39] Saraiva M. O’Garra A. (2010). The regulation of IL-10 production by immune cells. Nat. Rev. Immunol. 10 (3), 170–181. doi: 10.1038/nri2711 20154735

[B40] Schildknecht A. Brauer S. Brenner C. Lahl K. Schild H. Sparwasser T.. (2010). FoxP3+ regulatory T cells essentially contribute to peripheral CD8+ T-cell tolerance induced by steady-state dendritic cells. Proc. Natl. Acad. Sci. U. S. A. 107 (1), 199–203. doi: 10.1073/pnas.0910620107 20018763 PMC2806715

[B41] Schmidt A. Oberle N. Krammer P. H. (2012). Molecular mechanisms of treg-mediated T cell suppression. Front. Immunol. 3. doi: 10.3389/fimmu.2012.00051 PMC3341960 22566933

[B43] Tang C. L. Yang J. Cheng L. Y. Cheng L. F. Liu Z. M. (2017). Anti-CD25 monoclonal antibody enhances the protective efficacy of *Schistosoma japonicum* GST vaccine *via* inhibition of CD4+CD25+Foxp3+ regulatory T cells. Parasitol. Res. 116 (10), 2727–2732. doi: 10.1007/s00436-017-5581-0 28825137

[B44] Téllez-Martínez D. Batista-Duharte A. Portuondo D. L. Carlos I. Z. (2019a). Prophylactic and therapeutic vaccines against sporotrichosis. feasibility and prospects. Microbes Infect. 21 (10), 432–440. doi: 10.1016/j.micinf.2019.05.003 31201931

[B32] Téllez-Martínez D. Portuondo D. L. Loesch M. L. Batista-Duharte A. Carlos I. Z. (2019b). A Recombinant Enolase-Montanide™ PetGel A Vaccine Promotes a Protective Th1 Immune Response against a Highly Virulent Sporothrix schenckii by Toluene Exposure. Pharmaceutics 11 (3), 144. doi: 10.3390/pharmaceutics11030144 30934594 PMC6471120

[B45] Veldhoen M. Moncrieffe H. Hocking R. J. Atkins C. J. Stockinger B. (2006). Modulation of dendritic cell function by naive and regulatory CD4+ T cells. J. Immunol. 176 (10), 6202–6210. doi: 10.4049/jimmunol.176.10.6202 16670330

[B46] Wen Z. Wang X. Dong K. Zhang H. Bu Z. Ye L.. (2017). Blockage of regulatory T cells augments induction of protective immune responses by influenza virus-like particles in aged mice. Microbes Infect. 19 (12), 626–634. doi: 10.1016/j.micinf.2017.08.013 28899815 PMC5726911

[B47] Whibley N. Maccallum D. M. Vickers M. A. Zafreen S. Waldmann. H. Hori S.. (2014). Expansion of Foxp3(+) T-cell populations by *Candida albicans* enhances both Th17-cell responses and fungal dissemination after intravenous challenge. Eur. J. Immunol. 44 (4), 1069–1083. doi: 10.1002/eji.201343604 24435677 PMC3992851

