# Supplementary material for: Selective depletion of regulatory T cells enhances the immunogenicity of a recombinant-based vaccine against Sporothrix spp
Source: Front Cell Infect Microbiol. 2023 Feb 10;12:1084526. doi: 10.3389/fcimb.2022.1084526 (PMC9951613; doi:10.3389/fcimb.2022.1084526)
Supplement: Supplementary Figure 1 — Three aspects of primary lesions in the dorsal sacral region of mice infected with S. brasiliensis. (A) Suppurative ulcer; (B) Nodular lesion with a little central ulceration; (C) Nodular lesion. [file Presentation_1.pdf]

Supplementary Material

Selective depletion of regulatory T cells enhances the immunogenicity of a recombinant-based vaccine against *Sporothrix* spp.

Alexander Batista-Duharte*†, Damiana Téllez-Martinez†, Deivys Leandro Portuondo and Iracilda Zeppone Carlos*

Department of Clinical Analysis, School of Pharmaceutical Sciences, São Paulo State University (UNESP), Araraquara, SP, Brazil

Correspondence

Alexander Batista-Duharte (batista.duharte@unesp.br); Iracilda Zeppone Carlos (iracilda.zeppone@unesp.br)

Three aspects of primary lesions in the dorsal sacral region of mice infected with *S. brasiliensis*. A) Suppurative ulcer; B) Nodular lesion with a little central ulceration; C) Nodular lesion

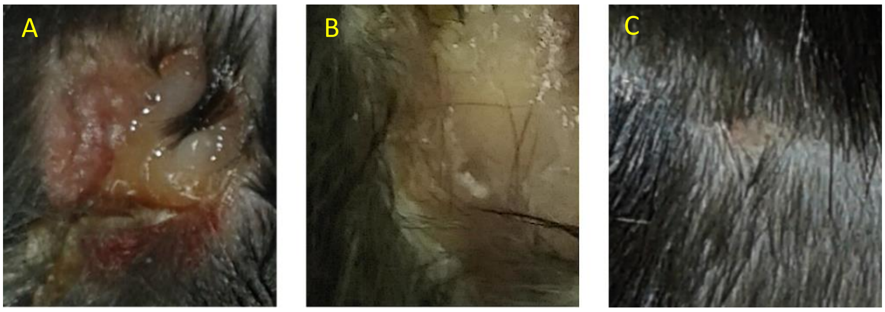

Main clinical findings in the *S. brasiliensis*- infected mice at 35 days post infection

	Non-vaccinated	rSSEno +G01	rSSEno +G01 Depleted 1st dose	rSSEno +G01 Depleted 2nd dose
**Suppurative ulcer**	5/7	2/7	1/7	1/7
**Nodular lesion with a little central ulceration**	2/7	2/7	3/7	2/7
**Nodular lesion**	0/7	3/7	3/7	4/7
**Secondary nodular lesions along the tail**	4/7	2/7	1/7	1/7
**Orchitis**	5/7	3/7	1/7	1/7
**Yellowish-white nodules in the liver***	6/7	3/7	2/7	2/7
**Death**	0/7	0/7	0/7	0/7

*In vaccinated mice the nodules in the liver were scarcely present, in a small number of animals. Morphological characteristic of the yellowish-white nodules in the liver of *S. brasiliensis*- infected mice, can be seen in (Batista-Duharte et al., 2018b)
